# NHS Health Checks: an observational study of equity and outcomes 2009–2017

**DOI:** 10.3399/BJGP.2020.1021

**Published:** 2021-06-15

**Authors:** John Robson, Cesar Garriga, Carol Coupland, Julia Hippisley-Cox

**Affiliations:** Institute of Population Health Sciences, Queen Mary University of London, London.; Nuffield Department of Primary Care Health Science, University of Oxford, Oxford.; Division of Primary Care, Faculty of Medicine and Health Sciences, University of Nottingham, Nottingham.; Nuffield Department of Primary Care Health Science, University of Oxford, Oxford.

**Keywords:** antihypertensives, cardiovascular disease, NHS Health Check, statins

## Abstract

**Background:**

The NHS Health Check cardiovascular prevention programme is now 10 years old.

**Aim:**

To describe NHS Heath Check attendance, new diagnoses, and treatment in relation to equity indicators.

**Design and setting:**

A nationally representative database derived from 1500 general practices from 2009–2017.

**Method:**

The authors compared NHS Health Check attendance and new diagnoses and treatments by age, sex, ethnic group, and deprivation.

**Results:**

In 2013–2017, 590 218 (16.9%) eligible people aged 40–74 years attended an NHS Health Check and 2 902 598 (83.1%) did not attend. South Asian ethnic groups were most likely to attend compared to others, and females more than males. New diagnoses were more likely in attendees than non-attendees: hypertension 25/1000 in attendees versus 9/1000 in non-attendees; type 2 diabetes 8/1000 versus 3/1000; and chronic kidney disease (CKD) 7/1000 versus 4/1000. In people aged ≥65 years, atrial fibrillation was newly diagnosed in 5/1000 attendees and 3/1000 non-attendees, and for dementia 2/1000 versus 1/1000, respectively. Type 2 diabetes, hypertension, and CKD were more likely in more deprived groups, and in South Asian, Black African, and Black Caribbean ethnic groups. Attendees were more likely to be prescribed statins (26/1000) than non-attendees (8/1000), and antihypertensive medicines (25/1000 versus 13/1000 non-attendees). However, of the 117 963 people with ≥10% CVD risk who were eligible for statins, only 9785 (8.3%) were prescribed them.

**Conclusion:**

Uptake of NHS Health Checks remains low. Attendees were more likely than non-attendees to be diagnosed with type 2 diabetes, hypertension, and CKD, and to receive treatment with statins and antihypertensives. Most attendees received neither treatment nor referral. Of those eligible for statins, <10% were treated. Policy reviews should consider a targeted approach prioritising those at highest CVD risk for face-to-face contact and consider other options for those at lower CVD risk.

## INTRODUCTION

Cardiovascular disease (CVD) remains the largest cause of premature mortality. CVD reduction has slowed in all Western countries, with widening socioeconomic gradients in the UK and, since 2018, the first increases in CVD mortality for 50 years.^1^^,^^2^ In 2009, the NHS Health Check 5-yearly programme started in England, with the aim of reducing heart attack and stroke, and identifying dementia in people aged 40–74 years by assessing major risk factors and providing individual behavioural support and treatment.^3^ In 2020, the programme completed its first decade, with coverage averaging 1 million annually. The effectiveness of the programme has been challenged by some researchers and clinicians.^4^^,^^5^ Uptake has been variable and low at <25%, contrasting with 50%–75% uptake in cancer screening. Half of the population have a low 10-year risk of CVD (<10%) for whom trials of health checks showed no evidence of reduced CVD, though new disease was identified.^5^^,^^6^ Whole population trials of behaviour change interventions for dietary and physical activity are difficult to implement and also lack evidence of CVD benefit.^7^^,^^8^ Evidence of whole population behavioural change thus relies on observational, epidemiological, and modelling studies or trials in high-risk subgroups.^9^^–^^11^ In contrast, pharmacological treatments or dietary substitution for hypertension or statins are supported by robust trial evidence.^12^^–^^14^

Public Health England has highlighted the need to address equity of provision and inequalities,^15^ and the aim of this current study was to compare NHS Health Checks from 2009–2013 with the period 2013–2017 and assess changes in attendance by age, sex, and ethnic and socioeconomic group, and new diagnosis of type 2 diabetes, hypertension, chronic kidney disease (CKD), dementia, and atrial fibrillation (AF), as well as new statin and antihypertensive treatment.^16^

## METHOD

The study conforms to the STROBE recommendations.^17^ The authors used QResearch, a nationally representative database including 35 million people registered with 1500 UK general practices using the same Egton Medical Information System (EMIS). The primary study period included the 4 years from 1 April 2013 to 31 March 2017 and the earlier study period 1 April 2009 to 31 March 2013. Adults aged 40–74 years registered for at least 1 year who were eligible for an NHS Health Check were included. Those excluded as ineligible had pre-existing hypertension, ischaemic heart disease, stroke or transient ischaemic attack, AF, heart failure, peripheral arterial disease, CKD, familial hypercholesterolaemia, or diabetes, were already on statins, or had had an NHS Health Check within 5 years before the study entry date.^18^ Data were extracted on 31 May 2018 using Read codes.^18^ Outcome data were obtained on or within 12 months of an NHS Health Check, or an index date in non-attendees of 1 April in the year of cohort entry. NHS Health Check attendance was based on specific codes and not imputed as it has been in some studies.^19^

**Table:** How this fits in

The uptake of NHS Health Check has remained low. Half of the attendees were <50 years or people at low CVD risk who received neither treatment nor referral. The NHS Health Check identified important new diagnoses of hypertension, type 2 diabetes, and chronic kidney disease, and this study reports increased new diagnoses of atrial fibrillation and dementia in people aged ≥65 years. Black African, Black Caribbean, and South Asian ethnic groups were more likely to be identified with disease compared to other ethnic groups. Treatment with statins was three times more likely in attendees than non-attendees, and treatment with antihypertensives was also increased. However, of those eligible for statins, only 8.3% were prescribed them. More targeted approaches should be considered to improve efficiency and cost effectiveness.

Sociodemographic data and risk factors were obtained on the closest date before or on the NHS Health Check or index date. Attendance was defined as attendees as a proportion of the eligible population in that year or period. The authors included sex, age group in years, and self-reported ethnic group using Office of National Statistics categories: White (British, Irish, and other White ethnic groups); South Asian (Bangladeshi, Indian, and Pakistani); Black African; Black Caribbean; Chinese; other, including mixed ethnic groups; and not recorded.^20^ Deprivation assessed by the Townsend score, derived at small area level from Census data on housing, car ownership, and unemployment,^21^ was grouped into fifths, with quintile 1 the least deprived and quintile 5 the most deprived.

Risk factors included smoking status, alcohol units per day, blood pressure, blood glucose, serum cholesterol, body mass index (BMI), and QRisk2 10-year cardiovascular risk, including a family history of ischaemic heart disease coded positive in first-degree relatives, with angina or heart attack <60 years. GP referrals for obesity, smoking, or alcohol reduction were recorded. Delays in diagnosis and treatment may occur due to repeat or detailed testing or referral.^22^ The authors therefore used a 12-month period after the NHS Health Check to allow completion of diagnostic and treatment processes. New diagnoses were recorded on or within 12 months of the NHS Health Check/index date, and included hypertension, CVD (ischaemic heart disease, or stroke or transient ischaemic attack), CKD, type 2 diabetes, AF, familial hypercholesterolaemia, and dementia. New diagnoses for hypertension and type 2 diabetes were based on practitioner-recorded disease register codes and not imputed from measurements. Diagnosis of CKD was based on national standards for estimated glomerular filtration (eGFR) values <60 ml/min/1.73 m^2^ (categories 3–5), and non-diabetic hyperglycaemia from HbA1c 42–47 mmol/mol.

New medication within 12 months was at least two statin prescriptions or two prescriptions out of the three main classes of antihypertensive medications of thiazide, calcium channel blocker, and angiotensin converting enzyme inhibitors or receptor blockers. ‘Not stated or recorded’ described missing values for ethnicity and deprivation.

A medical statistician used Stata MP (version 16) with Cox proportional hazards models to describe associations between outcomes and sociodemographic variables, using Royston-Parmar proportional hazards models when proportional hazard assumptions were not met. Models were adjusted for clustering by general practice, with calculated unadjusted and adjusted hazard ratios (HRs) and 95% confidence intervals (CIs) using a two-tailed test of significance of 0.01. HRs were adjusted for sociodemographic variables (see Supplementary Tables S1–S11).

## RESULTS

There were 5 518 796 potentially eligible people aged 40–74 years in the QResearch database from 1 April 2013 to 31 March 2017. Of these, 1 734 873 (31.4%) had ≥1 excluding conditions or treatments, leaving 3 783 923 people; from these, 291 107 (7.7%) were excluded as they had had an NHS Health Check within the previous 5 years. The study therefore comprised 3 492 816 eligible people, of whom 590 218 (16.9%) attended an NHS Health Check within 2013–2017 and 2 902 598 (83.1%) did not attend (see Supplementary Figure S1). The coverage of the 5-year rolling NHS Health Check programme, assuming attendance of one-fifth of the eligible population each year, is described in Table 1, increasing from 3.3% (19 001/572 766) in 2009 to 23.2% (139 587/602 129) in 2013; since then, it has remained stable, averaging 24.6% (590 218/2 400 157) over the 4 years 2013–2017.

**Table 1.** Coverage of NHS Health Check programme in each year, 2009–2017

**Primary study period 2013–2017**					**Secondary study period 2009–2013**				
**Financial year**	**Patients with Health Check in financial year, *n***	**Patients eligible in financial year, *n***	**20% of the eligible population, *n***	**% of coverage attendance one-fifth of eligible population**	**Financial year**	**Patients with Health Check in financial year, *n***	**Patients eligible in financial year, *n***	**20% of the eligible population, *n***	**% of coverage attendance one-fifth of eligible population**
**2013–2014**	139 587	3 010 646	602 129	23.2	**2009–2010**	19 001	2 863 831	572 766	3.3
**2014–2015**	156 745	3 007 610	601 522	26.1	**2010–2011**	49 589	2 891 798	578 360	8.6
**2015–2016**	147 763	2 996 987	599 397	24.7	**2011–2012**	96 919	2 889 866	577 973	16.8
**2016–2017**	146 123	2 985 545	597 109	24.5	**2012–2013**	135 466	2 866 188	573 238	23.6
**Total**	590 218	12 000 788	2 400 157	24.6	**Total**	300 975	11 511 683	2 302 337	13.1

### Demographic characteristics

Supplementary Table S12 describes attendees and non-attendees in 2013–2017 and 2009–2013. Attendance was 590 218 (16.9%) in 2013–2017 versus 300 975 (8.9%) in 2009–2013. In 2013–2017, more females attended (329 470/1 743 100, 18.9%) than males (260 748/1 749 716, 14.9%); 57.8% of females and 32.9% of males who attended were at low CVD risk (<5%) (see Supplementary Table S13).

Conversely, one in eight females and almost one in three males were at ≥10% CVD risk (see Supplementary Table S13). The largest group of attendees were aged <50 years, accounting for 286 559/590 218 (48.6%) NHS Health Check attendances in 2013–2017, and 132 590/300 975 (44.1%) of attendees in 2009–2013. Attendance increased with age and was 286 559/1 907 146 (15.0%), 177 627/976 164 (18.2%), 106 776/501 341 (21.3%), and 19 256/108 165 (17.8%) for age groups 40–49, 50–59, 60–69, and 70–74 years, respectively. In the earlier period, 2009–2013, there was no difference in attendance by deprivation quintile, but in 2013–2017 a greater proportion attended in less deprived than more deprived quintiles: Q1 133 493/750 828 (17.8%), Q2 131 539/733 089 (17.9%), Q3 118 238/714 534 (16.5%), Q4 103 569/671 062 (15.4%), and Q5 102 841/617 381 (16.7%), (see Supplementary Table S12). However, this was not significant after adjustment. In comparison with Q1, the adjusted HRs were Q4 HR 0.87 (95% CI = 0.82 to 0.93) and Q5 HR 0.89 (95% CI = 0.82 to 0.97) (see Supplementary Table S1). By ethnic group, patterns of attendance were similar in the two periods. Attendance in 2013–2017 was highest in Bangladeshi and Pakistani ethnic groups at 7221/18 695 (38.6%) and 9051/33 874 (26.7%), respectively, and lowest in Black African and Chinese ethnic groups at 12 917/60 688 (21.3%) and 3639/18 411 (19.8%), respectively (see Supplementary Tables S1 and S12).

### Risk factors by attendance status and by period

Table 2 shows CVD risk by attendance. Missing data for some risk factors were more likely in non-attendees. Major risk factor recording for attendees and non-attendees is described in Supplementary Table S14. In 2009–2013, 87 526/231 066 (37.9%) of attendees had ≥10% CVD risk, compared to 117 963/522 571 (22.6%) in 2013–2017. Supplementary Table S13 shows 104 166/427 717 (24.4%) of White attendees had a CVD risk of ≥10% in contrast to 328/11 210 (2.9%) among Black Africans in 2013–2017.

**Table 2.** CVD risk in people who did and did not attend for an NHS Health Check, recorded before or on the date of the NHS Health Check or relevant index date

**Primary study period 2013–2017**						**Secondary study period 2009–2013**					
	**Total eligible people, *n***	**Attendees, *n***	**%**	**Non-attendees, *n***	**%**		**Total eligible people, *n***	**Attendees, *n***	**%**	**Non-attendees, *n***	**%**
**Total**	3 492 816	590 218	—	2 902 598	—	**Total**	3 427 380	300 975	—	3 126 405	—
**QRisk2 recorded**	1 151 422	522 571	88.5	628 851	21.7	**QRisk2 recorded**	469 349	231 066	76.8	238 283	7.6
**QRisk2 not recorded**	2 341 394	67 647	11.5	2 273 747	78.3	**QRisk2 not recorded**	2 958 031	69 909	23.2	2 888 122	92.4
**<5%**	645 811	276 093	52.8	369 718	58.8	**<5%**	237 240	84 578	36.6	152 662	64.1
**5–9%**	276 870	128 515	24.6	148 355	23.6	**5–9%**	107 232	58 962	25.5	48 270	20.3
**10–14%**	128 159	63 629	12.2	64 530	10.3	**10–14%**	59 340	39 018	16.9	20 322	8.5
**15–19%**	62 682	32 466	6.2	30 216	4.8	**15–19%**	35 534	25 310	11.0	10 224	4.3
**≥20%**	37 900	21 868	4.2	16 032	2.5	**≥20%**	30 003	23 198	10.0	6805	2.9

*CVD = cardiovascular disease.*

Table 3 shows obesity (BMI ≥30 kg/m^2^) in 2013–2017 was more likely to be recorded in attendees (130 714, 22.1%) than non-attendees (407 409, 14.0%). Of these, 66 421 (50.8%) attendees, but only 18 352 (4.5%) of non-attendees, were referred to exercise programmes, and 70 803 (54.2%) and 14 444 (3.5%), respectively, to weight management. Current smokers comprised 90 741 (15.4%) of attendees and 576 888 (19.9%) of non-attendees; 74 866 (82.5%) of smoking attendees were referred to smoking cessation programmes compared to 210 453 (36.5%) of non-attendees. Current smokers in 2009–2013 comprised 53 503 (17.8%) of attendees. People drinking >6 units of alcohol per day in 2013–2017 comprised 29 703 (5.0%) of attendees compared to 24 715 (0.9%) recorded in non-attendees; of these heavier drinkers, 12 854 (43.3%) of attendees and 1311 (5.3%) of non-attendees were referred for alcohol management.

**Table 3.** Raised risk factors, referrals, and treatment for NHS Health Check attendees and non-attendees at or on index date or in the 12 months following

**Primary study 2013–2017**					**Secondary study 2009–2013**				
	**Attendees recorded, *n***	**% of recorded**	**Non-attendees recorded, *n***	**% of recorded**		**Attendees recorded, *n***	**% of recorded**	**Non-attendees recorded, *n***	**% of recorded**
**Raised risk factors**					**Raised risk factors**				
**Fasting glucose ≥7 mmol/L**	2085	0.9	4990	0.9	**Fasting glucose ≥7 mmol/L**	1988	1.5	8411	2.1
**Random glucose ≥11 mmol/L**	918	0.2	2500	0.3	**Random glucosee ≥11 mmol/L**	748	0.4	4030	0.4
**Raised BP: SBP ≥140 mmHg or DBP ≥90 mmHg**	150 652	25.6	216 728	9.6	**Raised BP: SBP ≥140 mmHg or DBP ≥90 mmHg**	88 847	29.6	316 602	12.9
**Obesity BMI ≥30 kg/m^2^ on or after NHSHC or index date**	115 201	19.7	88 674	4.3	**Obesity BMI ≥30 kg/m2 on or after NHSHC or index date**	56 944	19.2	135 595	6.1
**New referrals**					**New referrals**				
	**Attendees, *n***	**%**	**Non-attendees, *n***	**%**		**Attendees, *n***	**%**	**Non-attendees, *n***	**%**
**Current smokers up to NHSHC or index date**	90 741	15.4	576 888	19.9	**Current smokers up to NHSHC or index date**	53 503	17.8	641 882	20.5
**Current smokers referred to smoking cessation clinic**	74 866	82.5	210 453	36.5	**Current smokers referred to smoking cessation clinic**	43 295	80.9	212 121	33.0
**Obesity: BMI ≥30 kg/m^2^ up to NHSHC or index date**	130 714	22.1	407 409	14.0	**Obesity: BMI ≥30 kg/m^2^ up to NHSHC or index date**	66 630	22.1	414 733	13.3
**Weight referrals: BMI ≥30 kg/m^2^**	70 803	54.2	14 444	3.5	**Weight referrals: BMI ≥30 kg/m^2^**	34 524	51.8	22 578	5.4
**Exercise referrals: BMI ≥30 kg/m^2^**	66 421	50.8	18 352	4.5	**Exercise referrals: BMI ≥30 kg/m^2^**	36 952	55.5	24 639	5.9
**Alcohol>6 units/day up to NHSHC or index date**	29 703	5.0	24 715	0.9	**Alcohol >6 units/day up to NHSHC or index date**	6382	2.1	7775	0.2
**Alcohol referrals: >6 units/day**	12 854	43.3	1311	5.3	**Alcohol referrals:>6 units/day**	3668	57.5	613	7.9
**New treatment**					**New treatment**				
**≥2 prescriptions for statins**	15 470	2.6	23 450	0.8	**≥2 prescriptions for statins**	11 065	3.7	36 134	1.2
**≥2 prescriptions for antihypertensives**	14 461	2.5	38 745	1.3	**≥2 prescriptions for antihypertensives**	8508	2.8	49 790	1.6

*BMI = body mass index. BP = blood pressure. DBP = diastolic blood pressure. NHSHC = NHS Health Check. SBP = systolic blood pressure.*

**Figure 1.**
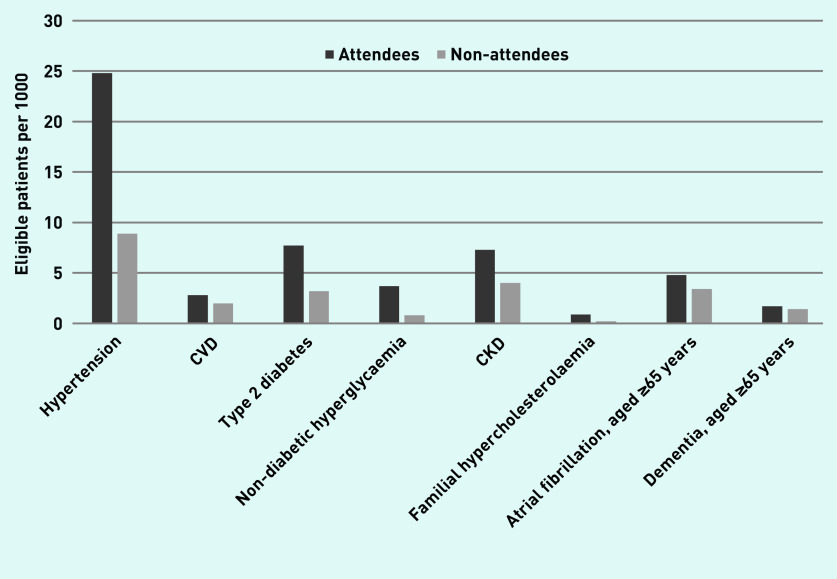
*New diagnoses in attendees and non-attendees 2013–2017 (at or in 12 months following the NHS Health Check or index date). CKD = chronic kidney disease. CVD = cardiovascular disease.*

Table 4 describes recording of risk factors and new diagnoses at or in the 12 months after the index date. Figure 1 shows new diagnoses in 2013–2017. For hypertension, new diagnoses in attendees were 25/1000 (one new case for every 40 people attending) versus 9/1000 in non-attendees; for type 2 diabetes 8/1000 (one new case for every 130 people) versus 3/1000; for CKD 7/1000 (one new case for every 138 people) versus 4/1000; for non-diabetic hyperglycaemia 4/1000 versus 1/1000; and for familial hypercholesterolaemia 0.9/1000 (one new case for every 1118 people) versus 0.2/1000. New diagnoses of AF in attendees aged ≥65 years was 5/1000 (one new case for every 209 people) versus 3/1000 in non-attendees, and for dementia it was 2/1000 (one new case for every 578 people) versus 1/1000, respectively. Adjusting for age, sex, and clustering by practice, new diagnoses were significantly more likely to be identified in attendees than non-attendees (*P*<0.001): hypertension HR 2.66 (95% CI = 2.51 to 2.81); CVD HR 1.34 (95% CI = 1.24 to 1.44); type 2 diabetes HR 2.35 (95% CI = 2.21 to 2.51); non-diabetic hyperglycaemia HR 4.11 (95% CI = 3.43 to 4.92); CKD HR 1.65 (95% CI = 1.52 to 1.78); familial hypercholesterolaemia HR 3.65 (95% CI = 3.15 to 4.21); AF HR 1.51 (95% CI = 1.31 to 1.74); and dementia HR 1.47 (95% CI = 1.17 to 1.84) (*P*<0.001) (see Supplementary Tables S2–S9). A hypertension diagnosis was more likely in Black African, Black Caribbean, Bangladeshi, ethnic groups other than White ethnic groups, and in more deprived quintiles (see Supplementary Table S2). Type 2 diabetes diagnosis was higher in all non-White ethnic groups (see Supplementary Table S3), with a gradient with increasing deprivation and a strong association with obesity (World Health Organization [WHO] obesity Class I BMI 30–34.9 kg/m^2^ adjusted HR 5.02, 95% CI = 4.45 to 5.65). Non-diabetic hyperglycaemia was higher in all non-White ethnic groups except Chinese, and in more deprived quintiles (see Supplementary Table S4).

**Table 4.** New diagnoses and risk factor recording for NHS Health Check attendees and non-attendees at or on index date or in 12 months following

**Primary study period 2013–17**						**Secondary study period 2009–13**					
	**Total eligible people, *n***	**Attendees, *n***	**%**	**Non-attendees, *n***	**%**		**Total eligible people, *n***	**Attendees, *n***	**%**	**Non-attendees, *n***	**%**
**Total**	3 492 816	590 218	—	2 902 598	—	**Total**	3 427 380	300 975	—	3 126 405	—
**New diagnoses**						**New diagnoses**					
**Hypertension**	40 439	14 616	2.5	25 823	0.9	**Hypertension**	40 519	7977	2.7	32 542	1.0
**CVD**	7394	1665	0.3	5729	0.2	**CVD**	8800	1111	0.4	7689	0.2
**Type 2 diabetes**	13 947	4555	0.8	9392	0.3	**Type 2 diabetes**	13 587	2486	0.8	11 101	0.4
**Non-diabetic hyperglycaemia**	4591	2157	0.4	2434	0.1	**Non-diabetic hyperglycaemia**	4158	1113	0.4	3045	0.1
**Chronic kidney disease**	15 993	4286	0.7	11 707	0.4	**Chronic kidney disease**	21 539	2537	0.8	19 002	0.6
**Familial hypercholesterolaemia**	1236	528	0.1	708	0.02	**Familial hypercholesterolaemia**	1448	306	0.1	1142	0.04
**Atrial fibrillation, aged ≥65 years**	1181	315	0.1	866	0.03	**Atrial fibrillation, aged ≥65 years**	1180	203	0.1	977	0.03
**Dementia, aged ≥65 years**	467	114	0.02	353	0.01	**Dementia, aged ≥65 years**	532	85	0.03	447	0.01
**Risk factor recording**						**Risk factor recording**					
**BMI**	790 847	502 427	85.1	288 420	9.9	**BMI**	698 709	244 641	81.3	454 068	14.5
**Positive family history premature CHD**	50 442	44 056	7.5	6386	0.2	**Positive family history of premature CHD**	30 757	20 668	6.9	10 089	0.3
**Blood pressure**	1 170 731	517 857	87.7	652 874	22.5	**Blood pressure**	1 111 009	260 577	86.6	850 432	27.2
**eGFR**	577 096	193 557	32.8	383 539	13.2	**eGFR**	531 056	97 746	32.5	433 310	13.9
**Fasting glucose**	164 838	59 588	10.1	105 250	3.6	**Fasting glucose**	229 876	54 585	18.1	175 291	5.6
**Random glucose**	281 151	124 916	21.2	156 235	5.4	**Random glucose**	343 042	89 632	29.8	253 410	8.1
**Total cholesterol**	651 654	379 727	64.3	271 927	9.4	**Total cholesterol**	538 034	186 389	61.9	351 645	11.2
**Cholesterol/HDL ratio**	583 505	347 723	58.9	235 782	8.1	**Cholesterol/HDL ratio**	441 177	179 642	59.7	261 535	8.4
**Smoking status**	1 073 223	503 438	85.3	569 785	19.6	**Smoking status**	1 090 038	251 633	83.6	838 405	26.8

*BMI = body mass index. CHD = coronary heart disease. CVD = cardiovascular disease. eGFR = estimated glomerular filtration rate. HDL = high density lipoprotein.*

New diagnosis of CKD was higher in Black Caribbean and Black African ethnic groups and in more deprived quintiles (see Supplementary Table S5). New CVD diagnosis was similar in attendees versus non-attendees (HR 1.06, 95% CI = 0.99 to 1.14). In attendees, Pakistani and Bangladeshi ethnic groups had higher risks of new CVD diagnosis and Black Africans lower risks, with an increasing gradient of CVD diagnosis with deprivation (see Supplementary Table S6). Familial hypercholesterolaemia is described in Supplementary Table S7. Atrial fibrillation and dementia diagnosis was more likely in males and at older ages 70–74 years; HRs are described in Supplementary Tables S8 and S9.

Table 3 shows statin treatment by period and attendance. In 2013–2017, new treatment with statins was more frequent among attendees (26/1000) than non-attendees (8/1000; HR 2.98, 95% CI = 2.84 to 3.13). Similarly, antihypertensive medicines were more likely to be prescribed in attendees (25/1000) than non-attendees (13/1000; HR 1.65, 95% CI = 1.59 to 1.72) (see Supplementary Tables S10 and S11).

Prescribing of statins and antihypertensives was higher in the earlier period because attendees were older and at higher risk than in the later period. In 2009–2013, statins were prescribed to 37/1000 and 12/1000 of attendees and non-attendees, respectively, and antihypertensives to 28/1000 and 16/1000, respectively. Supplementary Table S15 shows the gradient of increased statin prescriptions by category of CVD risk and attendance in the two study periods. Statin prescription was higher in the recorded risk categories in the later period, with the exception of those with <5% CVD risk.

Of those attendees eligible for statins with 10%–19% CVD risk, only 6.0% (5754/96 095) were treated and, of those at ≥20% CVD risk, 18.4% (4031/21 868) were treated. In 2013–2017, in people with a QRisk2 score ≥10%, statins were prescribed to 82.9/1000 and in 2009–2013 to 75.5/1000, with the later increase most pronounced in the group with risks of 10%–19%, at 38.9/1000 to 59.9/1000, respectively. South Asians were more likely and Black African, Black Caribbean, and Chinese ethnic groups were less likely to be prescribed statins than White ethnic groups (see Supplementary Table S10). Table 5 shows statin prescription by CVD risk.

**Table 5.** Statin prescription by CVD risk category in attendees

**CVD 10-year risk**	**Attendees, *n***	**Statin treated, *n***	**Attendees treated, %**	**% of all statin prescriptions**
**<5%**	276 093	1557	0.6	10.1
**5–9%**	128 515	2418	1.9	15.6
**10–19%**	96 095	5754	6.0	37.2
**≥20%**	21 868	4031	18.4	26.1
**Not recorded**	67 647	1710	2.5	11.1

*CVD = cardiovascular disease.*

## DISCUSSION

### Summary

This study provides new insights on equity of provision of NHS Health Checks, and new diagnosis and treatment by age, sex, and ethnic and socioeconomic group. Coverage increased initially in 2009–2013, but thereafter remained persistently low, averaging 24% over the years 2013–2017. Equity of attendance was variable, more likely in South Asian and less likely in Black African, Black Caribbean, and Chinese ethnic groups than White ethnic groups, and lower in males compared to females. In 2013–2017, people aged <50 years accounted for almost half of the attendances; 57.8% of females and 32.9% of males who attended were at low CVD risk (<5%). Conversely, one in eight females and almost one in three males were at ≥10% CVD risk.

Of those attendees eligible for statins with 10%–19% CVD risk, only 6.0% (5754/96 095) were treated and, of those at ≥20% CVD risk, 18.4% (4031/21 868) were treated. New statin treatment was almost three times more likely among attendees than non-attendees, and antihypertensive prescription was more likely in attendees. In attendees with CVD risk of 10%–19%, statin prescribing increased after revised 2014 National Institute for Health and Care Excellence guidance.^23^ South Asian ethnic groups (Indian, Pakistani, and Bangladeshi) were more likely and Black and Chinese ethnic groups were less likely to be prescribed statins than White ethnic groups. One new case of diagnosed hypertension was detected for every 40 people attending an NHS Health Check, one new case of type 2 diabetes for every 130 attendances; one new case of CKD for every 138 attendances; and one new familial hypercholesterolaemia case for every 1118 attenders. In attendees aged 65–74 years, one new case of AF was detected for every 209 patients and every 578 patients for dementia. Type 2 diabetes diagnosis was between three and six times more likely in South Asian than White ethnic groups, and CKD and hypertension were more likely in Black Caribbean and Black African ethnic groups. Increased diagnosis of these conditions was more likely in more socially deprived attendees, most pronounced with type 2 diabetes. At a public health level, increased diagnosis and treatment is likely to contribute equitably to the health needs of socially diverse populations.

Earlier detection of hypertension, type 2 diabetes, CKD, AF, and dementia was more likely in attendees. Attendees were almost three times more likely to be treated with statins and more likely to receive antihypertensive medication than non-attendees. For those at higher CVD risk, diagnosis and treatment confer important health benefits, but for those at low CVD risk, attendance lacks evidence of benefit.

### Strengths and limitations

GP payment for NHS Health Checks was based on specific codes, resulting in substantial coding completeness.^18^ NHS Health Checks provided by pharmacists or local authorities may not be recorded in GP records, but represent a small proportion of attendances.

Preventive programmes consistently report a healthy attendee effect, with substantial residual confounding in comparisons with non-attendees. Some studies propensity-matched attendees and non-attendees to reduce confounding.^19^ However, despite adjustment for all known confounders, the Danish Inter99 CVD prevention study showed non-attendees had substantially higher rates of accidental death and other unrelated causes than attendees.^41^^,^^42^ These biases reduce cardiovascular risks and events in attendees because they are likely to be healthier than non-attendees. The finding of higher rates of new diagnosis and treatment after NHS Health Checks runs counter to that bias and strengthens these findings.

### Comparison with existing literature

Other studies of NHS Health Checks have raised concerns about effectiveness, equity of delivery, and lack of benefit in people at low cardiovascular risk.^5^^,^^24^^,^^25^ A literature review of NHS Health Checks, largely before 2014,^24^ identified low coverage and poor lifestyle modification as issues of concern, a finding echoed by patients.^26^ The current study would indicate that little has changed. More than 75% of those eligible to attend do not do so. Though a referral may be recorded by GPs, there is no information on whether the patient attended. More than 80% of attendees did not receive a referral or treatment and added value was based on brief and often superficial advice from a healthcare assistant during a single visit taken up largely with recording.^25^^,^^27^

This study uptake, based on the registered GP population, was lower than national reporting, which used mid-year population estimates and invitation response as denominators.^28^ NHS Digital data on NHS Health Checks 2012–2018 did not include new diagnoses and treatments, and statin prescribing was almost twice as high as in this and previous studies indicating major methodological differences.^29^ No socioeconomic difference in attendance was reported.^29^

Like other earlier studies of 2009–2013,^16^^,^^30^^–^^35^ the present study of 2013–2017 identified more diagnoses of new hypertension, diabetes, and CKD, and higher prescription of statins among NHS Health Check attendees.^36^

This study is the first to report increased diagnoses of AF and dementia resulting from an NHS Health Check. Three previous studies considered AF, and none reported dementia, using populations comprised largely of people <65 years in which both conditions are rare and, hence, unlikely to identify changes in diagnosis.^36^^,^^37^ Chang *et al* found increased AF diagnosis in attendees, which was not significant after matching.^30^ The present study used a denominator of age 65–74 years followed for 12 months, and the authors observed significantly more new diagnoses of AF in attendees (5/1000) than non-attendees (3/1000), and new diagnoses of dementia were recorded in 2/1000 attendees versus 1/1000 non-attendees. Approximately 2% of people aged 65–74 years in community settings are estimated to have dementia.^38^ Lower attendance by Chinese and Black African patients was confirmed in the current study, which showed highest attendance in South Asians similar to reports in previous studies.^39^^,^^40^

### Implications for practice

Modelling of NHS Health Check effectiveness is a more appropriate method to estimate the CVD benefits of additional diagnoses and treatment, with estimates of 300 fewer premature deaths and 1000 more people living free of CVD.^43^^,^^44^ There is a policy decision to be made about whether it is more effective to improve persistently low attendance or target those at increased CVD risk.^45^ There is little evidence that attendees at lower CVD risk benefit from brief advice at NHS Health Checks and targeted approaches are more efficient.^46^^,^^47^

The COVID-19 pandemic halted the NHS Health Check programme in 2020, highlighting opportunities for online engagement for more than half of the population who have a CVD risk of <10% (Table 5). This would free up resources to improve targeting of people at higher CVD risk.^48^^,^^49^
